# Activation of sputter-processed indium–gallium–zinc oxide films by simultaneous ultraviolet and thermal treatments

**DOI:** 10.1038/srep21869

**Published:** 2016-02-23

**Authors:** Young Jun Tak, Byung Du Ahn, Sung Pyo Park, Si Joon Kim, Ae Ran Song, Kwun-Bum Chung, Hyun Jae Kim

**Affiliations:** 1 School of Electrical and Electronic Engineering, Yonsei University, 50 Yonsei-ro, Seodaemun-gu, Seoul 120-749, Republic of Korea; 2 Division of Physics and Semiconductor Science, Dongguk University, Seoul, 100-715, Korea

## Abstract

Indium–gallium–zinc oxide (IGZO) films, deposited by sputtering at room temperature, still require activation to achieve satisfactory semiconductor characteristics. Thermal treatment is typically carried out at temperatures above 300 °C. Here, we propose activating sputter- processed IGZO films using simultaneous ultraviolet and thermal (SUT) treatments to decrease the required temperature and enhance their electrical characteristics and stability. SUT treatment effectively decreased the amount of carbon residues and the number of defect sites related to oxygen vacancies and increased the number of metal oxide (M–O) bonds through the decomposition-rearrangement of M–O bonds and oxygen radicals. Activation of IGZO TFTs using the SUT treatment reduced the processing temperature to 150 °C and improved various electrical performance metrics including mobility, on-off ratio, and threshold voltage shift (positive bias stress for 10,000 s) from 3.23 to 15.81 cm^2^/Vs, 3.96 × 10^7^ to 1.03 × 10^8^, and 11.2 to 7.2 V, respectively.

Developments in flat panel display technology focus on greater display size, resolution, and flexibility. To achieve these goals with thin-film transistors (TFTs) as the display back-plane, excellent electrical characteristics and low fabrication temperatures are indispensable. For these reasons, oxide semiconductor (OS) TFTs are promising candidates for display back-plane devices. OS TFTs have higher transparency in the visible light range, higher mobility, and require lower deposition temperatures than amorphous Si TFTs^1^. However, even though IGZO films can be deposited on substrates at room temperature using RF sputtering, additional thermal treatments are needed for activation in order to obtain satisfactory semiconductor characteristics^2^. This is because ion bombardment during RF sputtering deposits metal-oxide compositions that are typically randomly deposited and generates structural defect sites related to oxygen vacancies^3^. Thermal treatments for OS activation are typically performed above 300 °C^4^. There are few published reports related to the activation of sputter-processed OS films, while studies related to solution-processed OS films using combustion methods through chemical synthesis^5^, high pressure annealing^6^ and microwave treatment^7^ have been extensively published. Low-temperature activation of sputter-processed OS films is needed in order to apply these materials to various flexible substrates having low melting temperatures and to reduce the thermal budget of the overall fabrication process. Here, we report an effective activation method for sputter-processed IGZO films at reduced temperatures using simultaneous ultraviolet and thermal (SUT) activation. SUT activation also enhanced the electrical characteristics and stability of the films. It was carried out after deposition of the IGZO films. This is different from our previous research, which is firstly reported on SUT treatment as the post treatment^8^. It is conducted after completely finished device fabrication with passivation layer to improve electrical performance of OS TFTs having already semiconductor characteristics. However, in this research, we focus that SUT treatment can be used for activation of IGZO film by changing UV wavelength from 365 nm to 185 nm and 254 nm, and temperature compared to previous research. We suggested mechanism of SUT activation by comparing various chemical and physical analyses in different ambient. (The detail explanation is shown in supplementary information) We believe that SUT method would significantly be interested in post treatment as well as activation source of sputter processed OS TFTs because it can be easily implemented by the display industry using the existing fabrication infrastructure because it does not require a vacuum chamber.

## Experimental Procedure

### Fabrication of IGZO TFTs on p^+^-Si substrate

Bottom-gate IGZO TFT structures were fabricated by depositing IGZO films onto a heavily-doped p^+^-Si substrate with a thermally-grown, 120-nm-thick SiO_2_ layer using an RF sputter system at room temperature. The heavily-doped p^+^-Si and 120-nm-thick SiO_2_ were used as the gate electrode and gate insulator, respectively. To fabricate 40-nm-thick IGZO films, RF power, working pressure, and oxygen partial pressure were set at 150 W, 5 mTorr and 0 Torr, respectively. The IGZO target consisted of In_2_O_3_:Ga_2_O_3_:ZnO at 1:1:1 mol%. SUT activation process was performed on the deposited IGZO films and consisted of sample irradiation with a mercury lamp-sourced UV light having a wavelength of 185 nm and 254 nm and a photon flux density of 60 mW/cm^2^, and thermal treatment at 50–300 °C on a hotplate for 1 h in air. We also performed only-thermal treatments at 50–300 °C for 1 h in air as a comparative study. SUT activation was also done in N_2_ for 1 h to exclude the effect of reactive oxygen radicals generated by the UV irradiation. Under optimized SUT activation conditions, we conducted only-UV, only-thermal, UV-after-thermal, and thermal-after-UV treatments to compare the effects of the different treatment sequences. Finally, 200-nm-thick Al electrodes were deposited by RF sputtering using a shadow mask. The width and length of the active layer were 1000 and 150 μm, respectively.

### Fabrication of IGZO TFTs on polyimide substrate

To explore the feasibility of SUT activation on flexible substrates, we deposited IGZO films on a polyimide (PI) film under the same conditions. The PI film, about 20 nm thick, was coated onto a carrier glass (alkali-free glass with a thickness of 0.5 mm) and then baked at 350 °C in air. Then, 100-nm-thick silicon nitride (SiN_x_) and 300-nm-thick silicon oxide (SiO_x_) bilayered buffer films were deposited over the entire substrate area by plasma enhanced chemical vapor deposition (PECVD) at 350 °C. These buffer films served as diffusion barriers to protect the PI film from ambient oxygen and moisture. A 200-nm-thick copper film was deposited as a gate electrode via RF sputtering and a 200-nm-thick SiO_2_ film was deposited as the gate insulator using PECVD. Then, the SUT activation process described above was used to activate the IGZO films. Finally, 200-nm-thick Al electrodes were deposited using RF sputtering with a shadow mask. The width and length of the active layers were 1000 and 150 μm, respectively.

### Electrical and physical measurements

The electrical characteristics of the activated TFTs were measured in the dark at room temperature using an HP4156C semiconductor parameter analyzer. To evaluate the positive bias stress (PBS) stability and positive bias temperature stress (PBTS), V_GS_ = 20 V and V_DS_ = 10.1 V were applied for 10,000 s in air at room temperature and 60 °C for the only-thermal and optimized SUT-activated TFTs, respectively. To further investigate the chemical characteristics after SUT activation, spectroscopic analyses were carried out using near-edge X-ray absorption spectroscopy (NEXAS), X-ray photoelectron spectroscopy (XPS), and spectroscopic ellipsometry (SE). NEXAS was used to examine the electronic structure near the conduction band of the SUT-activated IGZO films using the total electron yield (TEY) mode of the BL–10D beamline at the Pohang Accelerator Laboratory (PAL) in Korea. XPS analyzes were used to monitor quantitative and qualitative changes in surface composition, chemical structure, and valence band offset. SE was used to measure film density and optical bandgap by comparing complex refractive indices. Band alignments of the activated IGZO TFTs were established by combining the valence band offsets and optical bandgaps from the XPS and SE analyses, respectively.

## Results and Discussion

**Figure 1:**
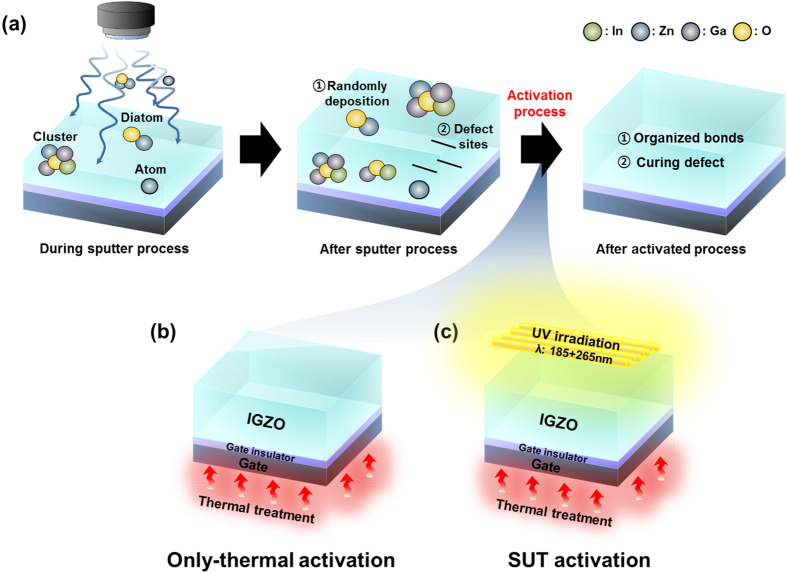
Fabrication of IGZO films using sputter processing. (**a**) Need for activation, (**b**) conventional thermal activation, and (**c**) SUT activation.

**Figure 2:**
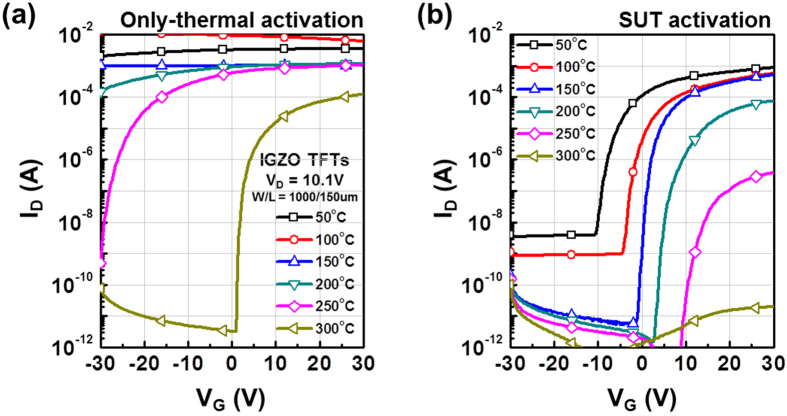
Transfer characteristics of (**a**) thermal and (**b**) SUT-activated IGZO TFTs as a function of temperature from 50–300 °C.

**Figure 3:**
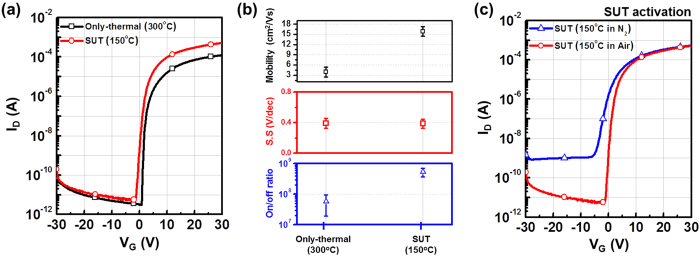
(**a**) Transfer characteristics of only-thermal (300 °C) and SUT (150 °C) activated IGZO TFTs. (**b**) Statistical parameters including mobility, S.S, and on/off ratio of only-thermal (300 °C) and SUT (150 °C) activated IGZO TFTs. (**c**) Transfer characteristics for IGZO TFTs that had been SUT activated in N_2_ or in air at 150 °C.

Figure 1(a) shows the conventional sputtering procedure used to fabricate IGZO films. IGZO films are typically deposited onto substrates into random states such as diatomic, atomic, and/or clusters^9^. Consequently, because of a lack of chemical bonds immediately after deposition, IGZO films tend to have multiple structural defect sites related to oxygen vacancies and interstitial cations. This is a general feature of ion bombardment^3^. For these reasons, to facilitate the organization of chemical bonds and reduce the number of defect sites, thermal treatment of the substrate is performed simultaneously with the sputtering process and/or activation is conducted after film deposition using a thermal treatment at temperatures above 300 °C (Fig. 1(b))^4^,^10^. However, in our research has shown that SUT activation, performed at lower temperatures, simultaneously improved the electrical characteristics and stability (Fig. 1(c)) of IGZO films. Figures 2(a,b) detail the transfer characteristics of the only-thermal and SUT-activated IGZO TFTs as a function of temperature from 50–300 °C, respectively. In the only-thermal case, the electrical behavior of the IGZO TFTs changed from metallic below 300 °C to semiconductor at 300 °C (Fig. 2(a)). This finding indicates that a temperature of at least 300 °C should be used to activate sputter-processed IGZO TFTs to achieve adequate semiconductor behavior, strong chemical bonds, and low numbers of defect sites. On the other hands, SUT-activated IGZO TFTs exhibited distinct semiconductor characteristics after processing at relatively low temperatures. In addition, their electrical behavior changed from semiconducting to insulating with increasing temperature (Fig. 2(b)). SUT treatment at 150 °C provided activated IGZO films having superior transfer characteristics. Thus, low-temperature processing is possible with SUT activation. Figure 3(a) compares the transfer characteristics for only-thermal and SUT-activated IGZO TFTs that were activated at 300 and 150 °C, respectively, and Fig. 3(b) shows statistical parameters including mobility (μ_FET_), sub-threshold swing (S.S), and on/off ratio of only-thermal and SUT-activated IGZO TFTs. As a result, The SUT-activated IGZO TFT exhibited superior transfer characteristics despite activation at a lower temperature. The effect of UV irradiation in air was also explored. SUT activation was performed in N_2_ to exclude the effect(s) of reactive oxygen radicals formed from ozone (Fig. 3(b)). UV irradiation at wavelengths of 185 and 254 nm can generate reactive oxygen radicals in air as follows^11^


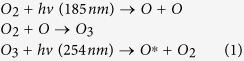


where O_2_ is molecular oxygen, O is mono-oxygen, O_3_ is molecular ozone, and O^*^ is a reactive oxygen radical. The energy of UV light at 185 and 254 nm is 6.7 eV and 4.8 eV, respectively. An energy of 6.7 eV is greater than the bond energy of O_2_ (5.13 eV), which means that molecular O_2_ would be decomposed to O. Then, O reacts with O_2_ to form O_3_ and thereby achieves a lower energy state. O_3_ could also decompose to O^*^ and O_2_ by exposure to 254-nm UV light. These generated O^*^ radicals would have higher diffusivity and reactivity at lower temperatures than would O_2_ and O. For these reasons, defect sites related to oxygen vacancies, interstitial metal cations, and carbon residues of surface would be effectively reduced by reacting the O^*^ in IGZO films^12^. However, with SUT activation in N_2_, the transfer characteristics of IGZO TFTs indicated imperfect activation despite the absence of O^*^ radicals. This finding implies that decomposition-rearrangement induced by the UV and thermal treatments assisted in the activation by controlling the formation of metal oxide (M–O) bonds in the IGZO films. These decomposition-rearrangement reactions may be comprised of UV-irradiation-induced dissociation of diatomic M–O bonds by the relatively high UV energy. The bond energies of Ga–O, In–O, and Zn–O in IGZO films are ca. 2.0 eV, ca. 1.7 eV and ca. 1.5 eV, respectively^8^. And then, decomposed atoms could rearrange during thermal treatment.

**Figure 4:**
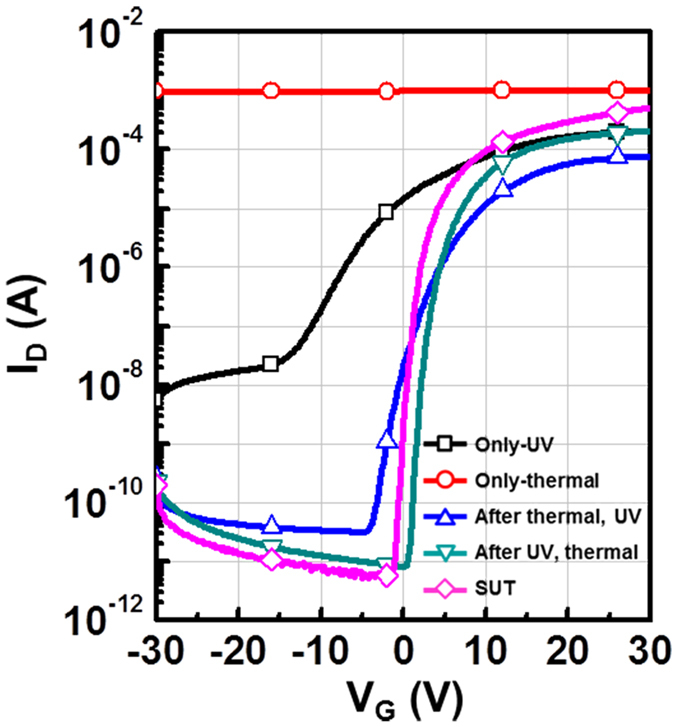
Transfer characteristics of activated IGZO TFTs made with different activation sequences: Only-UV, only-thermal, UV-after-thermal, thermal-after-UV and SUT.

**Figure 5:**
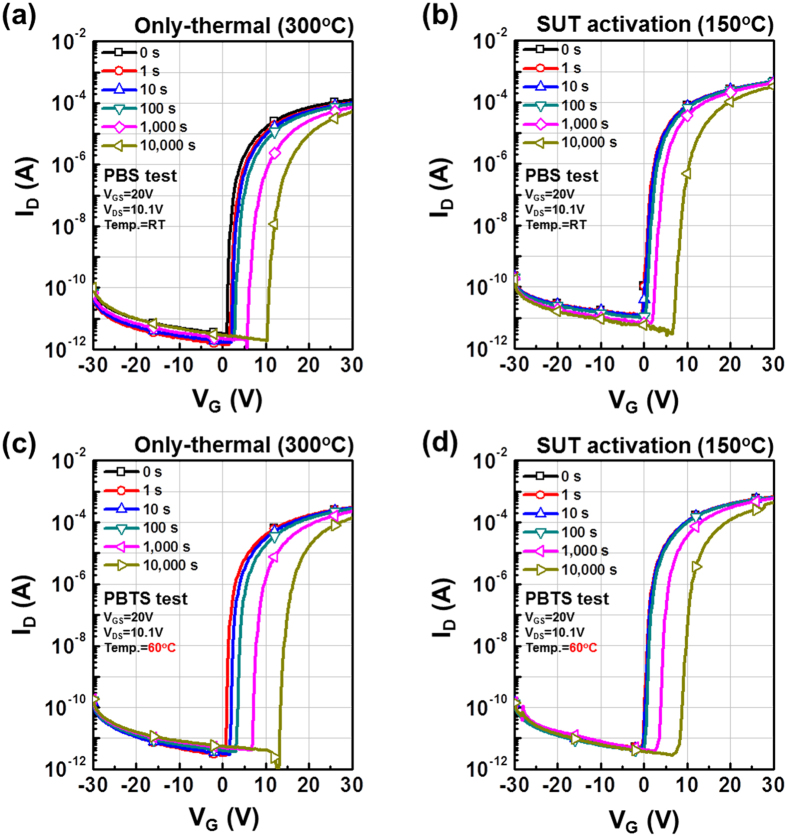
Variation in positive V_th_ shift, indicating the PBS and PBTS stability of only-thermal and SUT-activated IGZO TFTs as a function of stress time. (**a**) PBS and (**c**) PBTS stability of only-thermal activated IGZO TFTs, (**b**) PBS and (**d**) PBTS stability of SUT-activated IGZO TFTs.

**Figure 6:**
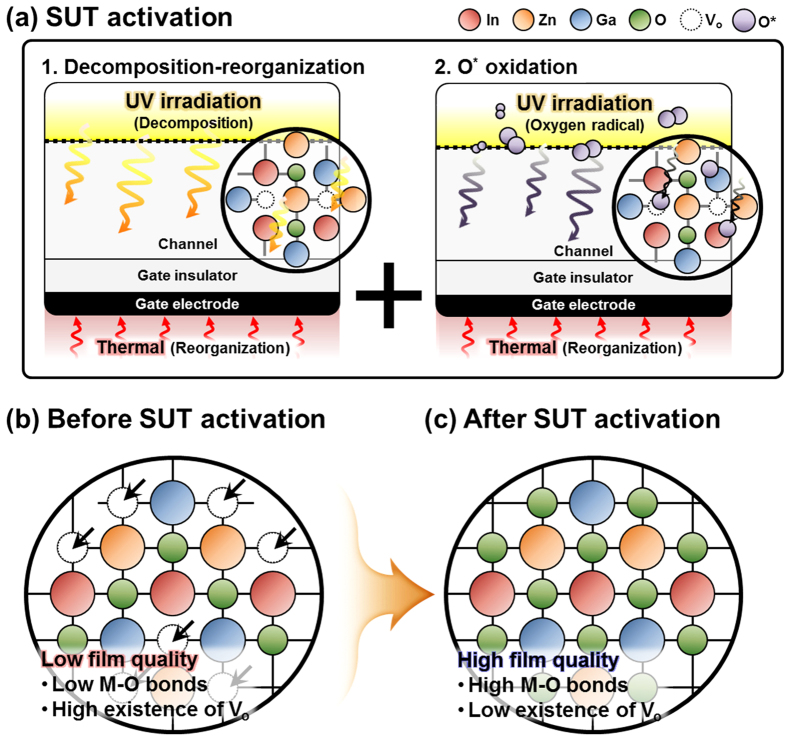
(**a**) Mechanism for SUT activation with simultaneous decomposition-reorganization and oxidation of O^*^ radicals in IGZO films. (**b**) Before SUT activation. (**c**) After SUT activation.

**Figure 7:**
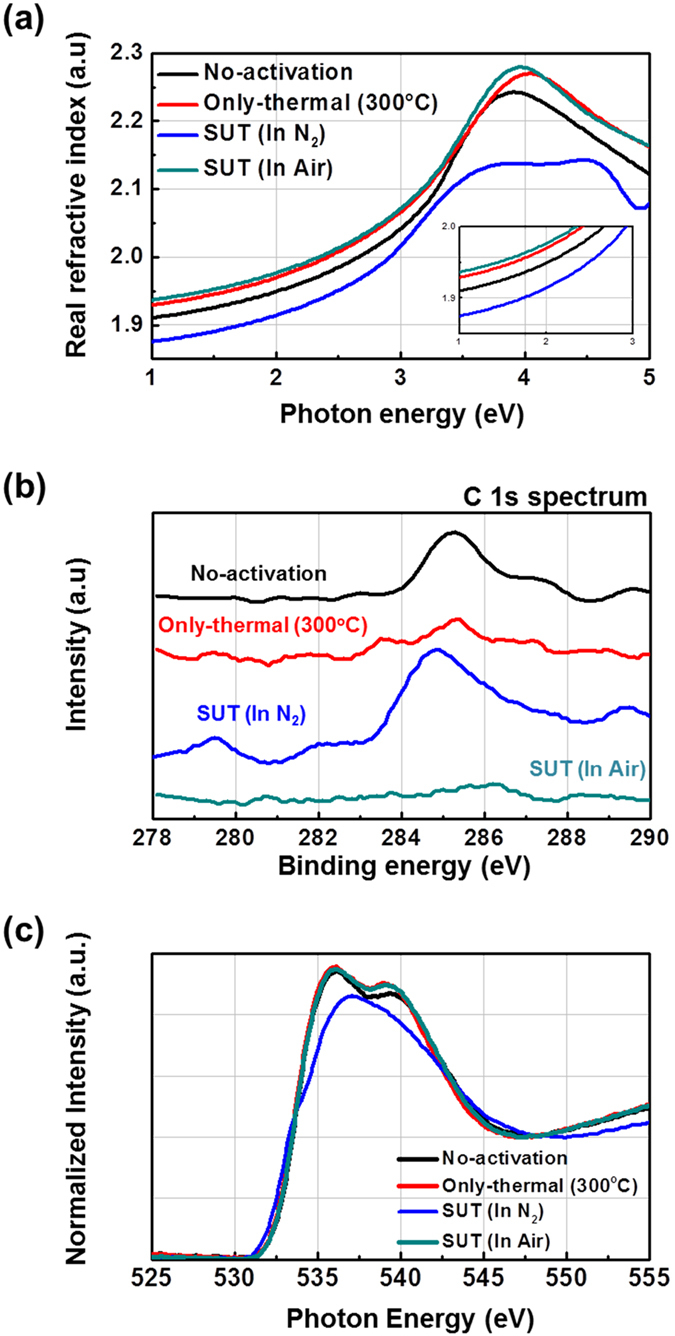
Variation of the (**a**) real refractive index, (**b**) C (1s) spectra and (**c**) O (1s) spectra of non-activated, only-thermal, SUT (in N_2_) and SUT (in air) activated IGZO films obtained using SE, XPS, and XAS analyses, respectively.

**Figure 8:**
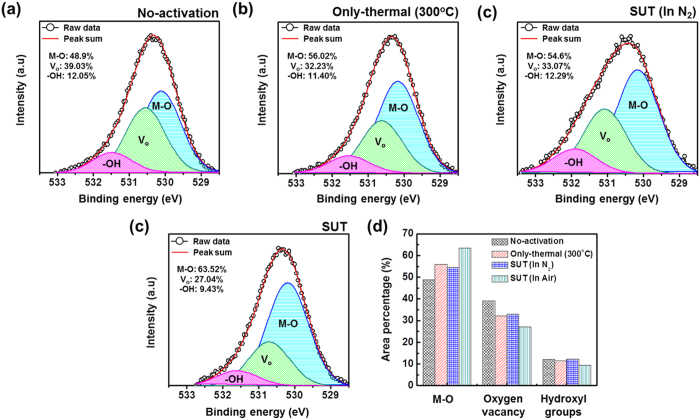
Variation of the O (1s) spectra of IGZO films after various activations: (**a**) Non-activated, (**b**) only-thermal, (**c**) SUT (in N_2_) and (**d**) SUT (in air). (**d**) Comparison of the peak area percentages for M–O, V_o_, and hydroxyl groups from the deconvolution of the O (1s) spectra.

**Figure 9:**
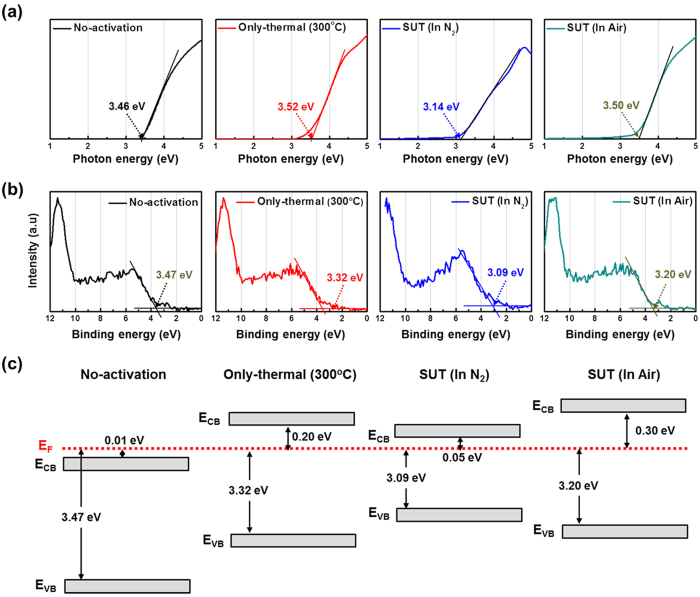
Variation of (**a**) optical band gap and (**b**) valence band offset spectra for non-activated, only-thermal, SUT (in N_2_) and SUT (in air) activated IGZO films using SE and XPS analyses, respectively. (**c**) Design of band alignment in non-activated, only-thermal, SUT (in N_2_) and SUT (in air) activated IGZO films by combining the optical band gap and the valence band offset data.

**Figure 10:**
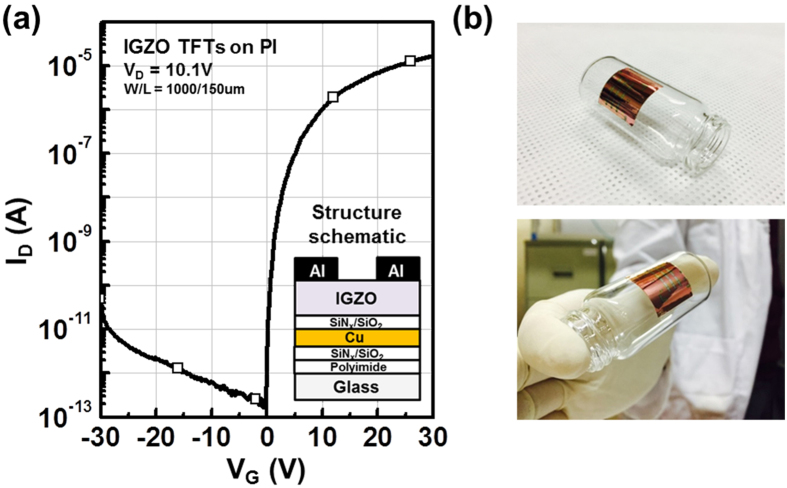
(**a**) Transfer characteristics of SUT-activated IGZO TFTs on PI substrates. (**b**) Photograph of an IGZO TFT on a PI substrate.

Therefore, to achieve stable transfer characteristics, SUT activation should be conducted in air in order to simultaneously react O^*^ radicals and promote decomposition-rearrangement. Figure 4 shows the transfer characteristics obtained using various activation conditions, including only-UV, only-thermal, UV-after-thermal, thermal-after-UV, and SUT. These conditions were selected to evaluate the individual effects and treatment sequences for the optimization of the SUT activation (at 150 °C and with UV irradiation for 1 h). Table 1 lists the electrical parameters of μ_FET_, on/off ratio, S.S, and maximum trapped charge density (N_max_). Of the synthesized TFTs, the SUT-activated TFT had superior electrical characteristics. However, Fig. 4 shows that TFTs treated with only-UV and only-thermal conditions were not activated. This is because decomposition of M–O bonds occurs without rearrangement during thermal treatment, and rearrangement occurs without decomposition of M–O bonds and the reaction of O^*^ radicals during UV irradiation. For the UV-after-thermal and thermal-after-UV treatments, we believe that sequential treatment would be insufficient for activation. The decomposition of M–O bonds induced by the UV irradiation would rapidly and undesirably occupy the low energy states via structural relaxation over the exposure time^8^. Therefore, thermal treatment should be performed concurrently with UV irradiation to establish strong chemical bonds. The PBS and PBTS stability of SUT-activated and only-thermal (300 °C) activated TFTs was also evaluated. PBS and PBTS measurements were done at V_GS_ = 20 V and V_DS_ = 10.1 V for 10,000 s at room temperature and at 60 °C, respectively (Fig. 5). The V_th_ of the only-thermal and SUT-activated TFTs were shifted by 11.2 and 7.2 V under PBS test and by 13.5 and 8.5 V under PBTS test, respectively. From the unchanged SS values measured for both the only-thermal and SUT-activated TFTs, we can exclude the defect creation model but should consider two alternative models: (1) Trapped negative charges at the active layer/gate insulator interface^12^ and (2) the adsorption of molecules from the environment at the back-active surface^13^. SUT activation would effectively reduce the contributions from these two models. First, SUT activation enhances film quality by increasing the number of M–O bonds and decreasing the number of defect sites related to oxygen vacancies and carbon residues of surface by simultaneously inducing decomposition-rearrangement and reacting O^*^ radicals in the IGZO films^13^. The trapping of negative charges in the IGZO films are thereby reduced. Second, generated O^*^ radicals are rigidly adsorbed onto the back-active surface and are converted to OH species by interactions with air^14^. Thus, additional adsorption of molecules at the back-active surface becomes effectively blocked. The chemical mechanism of SUT activation is illustrated in Fig. 6. Simultaneous usage of 185- and 254-nm UV light and thermal treatment at 150 °C in air serves to decompose those M–O bonds having bond energies lower than the energy of the impingent light. This process also generates O^*^ radicals. The concurrent thermal treatment not only induces reorganization of the decomposed M–O bonds but also promotes the reaction of O^*^ radicals with defect sites, including oxygen vacancies, interstitial cations, and carbon residues of surface. Therefore, SUT activation improved film quality by increasing the number of M–O bonds and decreasing the number of defect sites. To confirm the electrical characteristics and proposed chemical mechanisms, SE and XPS analyses were conducted on non-activated, only-thermal (300 °C), SUT (in N_2_ at 150 °C and in air at 150 °C) activated IGZO films. Figure 7(a) shows the real refractive index (R-value) derived from the SE spectra, which indicates film density as a function of photon energy. A high R-value indicates a denser film^15^. The IGZO film that had been SUT-activated in air had the highest R-value, while that activated in N_2_ had the lowest R-value. This result indicates that the number of M–O bonds were fewer after SUT activation in N_2_ than after SUT activation in air because of the absence of O^*^ radical reactions under the former condition. Furthermore, the M–O bonds decomposed by UV irradiation could not re-arrange sufficiently at 150 °C because the IGZO film that had been SUT-activated in N_2_ had a lower film density than the non-activated IGZO film. Figure 7(b) shows XPS C (1s) spectra of the activated IGZO films after Ne ion surface sputtering for 1 min before XPS measurement to eliminate carbon contamination. However, although surface sputtering was performed, some carbon residues could be remained on film surface depending on the activation process. As a result, the film that had been SUT-activated in air had relatively few remnant carbon contamination on the surface, comparable to the only-thermal activated film, because surface remnant carbon were significantly reduced through reactions with O* at both low and elevated temperatures. For these reasons, films that were not activated or SUT-activated in N2 would be relatively inefficient at eliminating the carbon residues from IGZO surfaces. XAS was used to investigate the electronic structure near the conduction band (Fig. 8). XAS probes unoccupied states related to charge transport in the conduction band. The spectra were normalized by subtracting the X-ray beam background and scaling of post-edge level in the raw data. Through this normalization, qualitative changes could be assessed for the respective activation types. The normalized oxygen K_1_ edge spectra of IGZO correspond to the orbital hybridizations of In_2_O_3_, Ga_2_O_3_, and ZnO, which are directly related to the oxygen p-projected states of In 5*sp* + O 2*p*, Ga 4*sp* + O 2*p*, and Zn 4*sp* + O 2*p*, respectively^16^. The observed XAS features were similar to those reported elsewhere for IGZO films except for the SUT-activated IGZO film in N_2_. This means that the IGZO film that had been SUT-activated in N_2_ had a conduction band electronic structure differing from those of the other activated IGZO films, which also exhibited different film densities and amounts of carbon residue. Another interesting feature concerned the ordered orbital structures of the only-thermal and SUT-activated IGZO films in air. The ZnO orbital near 540 eV was enhanced, which led to a larger conduction band through an increase in the number of unoccupied states. XPS O (1*s*) spectra were examined to characterize changes in the chemical oxygen composition (Fig. 8). The spectra of activated IGZO films were deconvoluted using Gaussian distributions after correcting for the background. This was necessary because of lattice-related metal oxide (M–O) and non-lattice-related oxygen vacancies (V_o_) and hydroxyl groups (–OH) present in the OS films^17^. Three different O (1*s*) peaks were observed at 530.3 ± 0.2, 531.2 ± 0.2, and 532.1 ± 0.2 eV. The mechanism proposed in Fig. 6 can be used to understand these spectra. The peaks corresponding to M–O bonds and V_o_ had the highest and lowest area percentages in the spectra of IGZO films that had been SUT-activated in air. Therefore, SUT activation in air effectively achieved high film quality at a lower temperature than that required for only-thermal activation. In contrast, the non-activated film showed the opposite characteristics: V_o_ and M–O had the highest and lowest area percentages in the O (1s) spectrum. This implies that the transfer characteristics of non-activated IGZO TFT were metal-like because of the high carrier concentration caused by V_o_. Although the IGZO film that had been SUT-activated in N_2_ had a better film quality than the non-activated film, the M–O bonds dissociated by UV light may not have been completely rearranged because of insufficient thermal treatment at 150 °C and the absence of O^*^ radical reaction. Therefore, to allow complete decomposition-rearrangement and reaction with O^*^ radicals, SUT activation should be performed simultaneously in air or O_2_. Figure 9 shows the band alignment for activated IGZO films. Optical band-gap energies and valence band offsets were derived from SE and XPS data, respectively, and are shown in Fig. 9(a,b). These results are consistent with a change in the carrier concentration across the Fermi energy level contributed by V_o_ between the conduction and valence bands^18^. Figure 9(c) shows that the Fermi levels of non-activated IGZO films and IGZO films that had been SUT-activated in N_2_ were located above and near the conduction band, respectively. These treated IGZO TFTs had metallic electrical characteristics with high off-currents and negative V_th_ shifts. For the only-thermal and SUT activations in air, the Fermi level was located relatively far from the conduction band, resulting in semiconductor characteristics. This means that only-thermal and SUT activation in air were more effective at activating the IGZO films. However, of these two activation types, the only-thermal activated IGZO film contains more V_o_ than the IGZO films that had been SUT activated in air. That is because the conduction band offset of the only-thermal treated film was lower than that of the film that had been SUT-treated in air. In other words, because of the abundance of V_o_, which are defect sites, the only-thermal activated IGZO TFTs exhibited poorer electrical performance and PBS stability than did the films that had been SUT-treated in air^19^. Finally, to confirm the applicability of SUT activation for flexible substrates, we conducted SUT activation on an IGZO TFT that had been fabricated on a PI substrate. The transfer characteristics of this TFT fluctuated more than those made on p^+^-Si substrates because of the different materials and deposition conditions used to form the gate insulator and gate electrode (Fig. 10(a)). Nevertheless, the data indicate that SUT activation on a flexible substrate is feasible. Figure 10(b) is a photograph of an IGZO TFT fabricated on the PI substrate. Relative to only-thermal activation, SUT activation of IGZO films permits low-treatment processing, i.e., 150 °C, and simultaneously enhances the electrical characteristics and stability. The SUT treatment described herein increases the number of M–O bonds that act as current paths and reduces the number of defect sites associated with oxygen vacancies.

## Conclusion

SUT activation of sputter-processed IGZO TFTs was closely examined. An activation temperature was 150 °C improved the electrical characteristics and PBS stability compared with only-thermal activation. Various treatment sequences were evaluated for UV and thermal activation. It was established that UV irradiation and thermal treatment should be carried out simultaneously to provide activated IGZO TFTs with the best electrical characteristics. SUT activation effectively increased M–O bond formation and decreased the number of defect sites such as oxygen vacancies and carbon residues of surface. This resulted in high-quality films. A chemical mechanism proposed for SUT activation, including simultaneous decomposition-rearrangement and reaction with O^*^ radicals, was derived from the electrical characteristics and XPS and SE spectroscopic data. The SUT-activated IGZO TFT exhibited better electrical performance than the only-thermal activated film. The μ_FET_ increased from 3.23 to 15.81 cm^2^/Vs, the on-off ratio increased from 3.96 × 10^7^ to 1.03 × 10^8^, and the positive V_th_ shift (an indicator of PBS stability) decreased from 11.2 V to 7.2 V. This research should be straightforward to implement by the display industry and provides various options for flexible substrates.

## Additional Information

**How to cite this article**: Tak, Y. J. *et al.* Activation of sputter-processed indium-gallium-zinc oxide films by simultaneous ultraviolet and thermal treatments. *Sci. Rep.* **6**, 21869; doi: 10.1038/srep21869 (2016).

## Supplementary Material

Supplementary Information

## Figures and Tables

**Table 1:** Summary of electrical parameters such as mobility, on/off ratio, SS, and N_max_ of only-UV, only-thermal, UV-after-thermal, thermal-after-UV, and SUT activated devices.

Sample	Mobility (cm^2^/Vs)	S.S (V/dec)	On/off ratio	N_it_
Only-UV	3.58	3.85	3.83 × 10^4^	1.05 × 10^13^
Only-thermal	—	—	—	—
UV-after-thermal	3.05	1.27	2.48 × 10^6^	3.37 × 10^12^
thermal-after-UV	8.95	0.57	2.47 × 10^7^	1.45 × 10^12^
SUT	15.81	0.54	1.03 × 10^8^	1.33 × 10^12^
