# Supplementary material for: Activation of sputter-processed indium–gallium–zinc oxide films by simultaneous ultraviolet and thermal treatments
Source: Sci Rep. 2016 Feb 23;6:21869. doi: 10.1038/srep21869 (PMC4763207; doi:10.1038/srep21869)
Supplement: Supplementary Information [file srep21869-s1.pdf]

Supplementary Information for

Activation of sputter-processed indium–gallium–zinc oxide films by simultaneous ultraviolet and thermal treatments

Young Jun Tak[1, ‡], Byung Du Ahn[1, ‡], Sung Pyo Park[1], Si Joon Kim[1], Ae Ran Song[2], Kwun-Bum Chung[2,★], Hyun Jae Kim[1,★]

[1]*School of Electrical and Electronic Engineering, Yonsei University, 50 Yonsei-ro, Seodaemun-gu, Seoul 120-749, Republic of Korea*

[2]*Division of Physics and Semiconductor Science, Dongguk University, Seoul, 100-715, Korea*

★E-mail: kbchung@dongguk.edu, hjk3@yonsei.ac.kr

‡Y. J. Tak and B. D. Ahn contributed equally to this work.

Comparison "SUT activation" with "previous SUT treatment"

We firstly reported SUT treatment as the "POST TREATMENT" to improve electrical performance and stability on IGZO TFTs in [ACS Appl. Mater. Interfaces, 6, 6399-6405, 2014]. However, it is completely different as a view point of "purpose, methods, process steps, and mechanism" compared to this research.

For purpose, previous research was focused on "POST TREATMENT",[1] which is conducted after deposition of passivation layer. Before the SUT treatment, IGZO TFTs have already satisfactory semiconductor characteristics, but semiconductor characteristics could be improved after SUT treatment. In contrast, in this research, we focused on "ACTIVATION" in order to change IGZO films from metallic to semiconductor characteristics. Sputter-processed IGZO films were generally activated using only-thermal treatment above 300°C by improving chemical bonds from weak- to well- and reducing sub-gap states caused by oxygen.[2] However, this temperature is obstacle to choose various flexible substrates having low melting temperature. Therefore, by modifying experimental conditions compared to previous research, SUT activation can decrease temperature from 300°C to 150°C with improved electrical performance and stability.

For methods, in the previous research, SUT treatment was simultaneously performed as the post treatment using UV system with wavelength of 365nm and thermal treatment at 200°C.[1] We found optimized condition of SUT treatment for post treatment through various experimental splits. However, in this research, if we used SUT treatment for activation as the same condition of previous research (UV irradiation (365nm) + thermal (200°C)), IGZO films was not activated to get satisfactory semiconductor characteristics as shown in figure S1. Therefore, to utilize SUT treatment as the activation source, wavelength of UV system should be modified as the 185nm and 254nm. In this research, we successfully found optimized condition of SUT treatment for activation source.

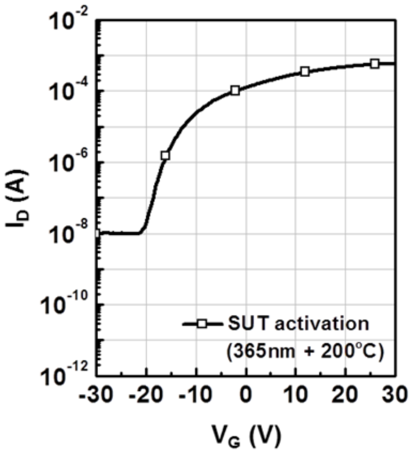

Figure S1. Transfer characteristics of SUT activated IGZO TFT
(SUT condition: UV irradiation (365nm) + thermal (200°C))

For process steps, as mention above, SUT treatment in previous research was conducted as the post treatment, which is conducted after "completely finished device fabrication with passivation layer". Furthermore, before the SUT treatment, IGZO TFTs already have semiconductor characteristics. However, in this research, we conducted SUT activation "after deposition of IGZO film without passivation". In additional, before the SUT activation, IGZO TFTs do not have semiconductor characteristics, and they have semiconductor characteristics after the SUT activation.

For mechanism, in this research, we used UV system with wavelength of 185nm and 254nm compared to previous research. Therefore, in SUT activation, we should consider two effects including "decompose-rearrangement and oxidation through oxygen radical". Previous research only considered the "decomposition-rearrangement". Therefore, to investigate two effects in SUT activation, we conducted physical and chemical analysis in different ambient such as N_2 and air.

For these reasons, this research is completely different from previous research. Figure S2 illustrate comparison of "POST TREATMENT" and "ACTIVATION" using SUT treatment.

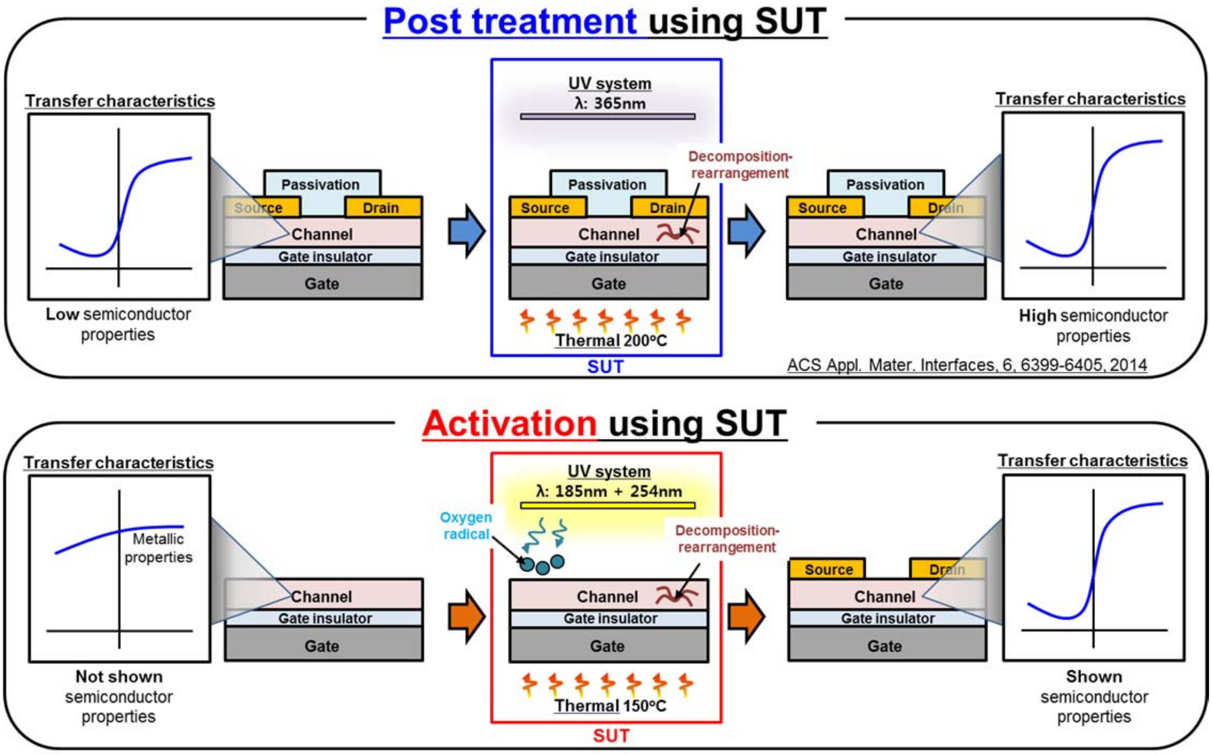

Figure S2. Comparison of post treatment and activation using SUT treatment

Reference

[1] Tak, Y. J. et al. Enhanced Electrical Characteristics and Stability Via Simultaneous Ultraviolet and Thermal Treatment of Passivated Amorphous In–Ga–Zn–O Thin-Film Transistors. ACS applied materials & interfaces. 6, 6399-6405 (2014)

[2] Kuo, S.-Y. et al. Effects of RF power on the structural, optical and electrical properties of Al-doped zinc oxide films. Microelectronics Reliability. 50, 730-733 (2010)
